# Characterization of neurons in the primate medial intraparietal area reveals a joint representation of intended reach direction and amplitude

**DOI:** 10.1371/journal.pone.0182519

**Published:** 2017-08-09

**Authors:** Rishi Rajalingham, Sam Musallam

**Affiliations:** 1 Department of Brain & Cognitive Sciences, Massachusetts Institute of Technology, Cambridge, Massachusetts, United States of America; 2 Department of Electrical & Computer Engineering, McGill University, Montreal, Canada; University of Muenster, GERMANY

## Abstract

To support accurate memory-guided reaching, the brain must represent both the direction and amplitude of reaches in a movement plan. Several cortical areas have been shown to represent the direction of a planned reaching movement, but the neuronal representation of reach amplitude is still unclear, especially in sensory-motor integration areas. To investigate this, we recorded from neurons in the medial intraparietal area (MIP) of monkeys performing a variable amplitude memory reach task. In one monkey, we additionally recorded from the dorsal premotor cortex (PMd) for direct cross-area comparisons. In both areas, we found modest but significant proportions of neurons with movement-planning activity sensitive to reach amplitude. However, reach amplitude was under-represented relative to direction in the neuronal population, with approximately one third as many selective neurons. We observed an interaction between neuronal selectivity for amplitude and direction; neurons in both areas exhibited significant modulation of neuronal activity by reach amplitude in some but not all directions. Consistent with an encoding of reach goals as a position in visual space, the response patterns of MIP/PMd neurons were best predicted by 2D Gaussian position encoding model, in contrast to a number of alternative direction and amplitude tuning models. Taken together, these results suggest that amplitude and direction jointly modulate activity in MIP, as in PMd, to form representations of intended reach position.

## Introduction

Humans and non-human primates are capable of accurately reaching to the remembered location of a visual target [1]. We are all familiar with the seemingly simple act of reaching for a coffee cup while maintaining our gaze on the words on a page. To support this “memory-guided” reaching behavior, the primate brain must be capable of extracting information about both the *direction* and *amplitude* of the reach movement goal, and encoding this information in a motor plan. Several decades of research in non-human primates has shown that reach direction is well represented in motor planning signals in several cortical areas; the direction of a planned movement modulates the spiking activity of neurons in posterior parietal sensory-motor integration areas (V6A, MIP, area 5 and 7a), premotor (PMd) and motor (M1) cortices [2–10]. Similarly, many studies have examined the representation of reach depth in both association and motor and sensory-motor integration areas [11–15]. In contrast, similar evidence for neuronal modulation by reach amplitude in the fronto-parallel plane is largely restricted to motor output areas PMd and M1 [4, 16–21], while higher-level association areas have not yet been examined using neurophysiological techniques. A recent functional MRI adaptation study in humans reported that posterior parietal cortex and frontal regions responded differentially to reach amplitude [22]. Since this non-invasive method offers limited temporal resolution, these differences could not be attributed to movement planning signals, and direct physiological data addressing this question is still lacking. Here, we directly tested the hypothesis that the movement planning neuronal activity in areas specialized for reaching in the posterior parietal cortex represents both the direction and amplitude of a planned reach.

To this end, we recorded from neurons in the medial intraparietal area (MIP) from three monkeys while they performed a variable amplitude memory reach task. MIP, a sub-area of the parietal reach region (PRR) [23], is located on the medial bank of the intraparietal sulcus, receives inputs from nearby visual and proprioceptive areas [24–27] and, importantly, is specialized for reaching movements [28]. We also simultaneously recorded from the dorsal premotor cortex (PMd) in one of the three monkeys for direct cross-area comparisons. We found that a modest but statistically significant proportion of neurons in both MIP and PMd modulated their movement planning activity based on reach amplitude. In both areas, we observed conjoint encoding of reach direction and amplitude, with significant modulations of neuronal activity by reach amplitude in some but not all reach directions. Finally, we found that a Gaussian target-position encoding model better predicted the neuronal movement planning responses in both areas, compared to alternative models of direction and amplitude tuning. Taken together, these results suggest that neurons in MIP, as in PMd, may jointly encode amplitude and direction signals to form a high-level representation of intended reach target position.

## Materials and methods

### Subjects and preparatory surgery

**Fig 1:**
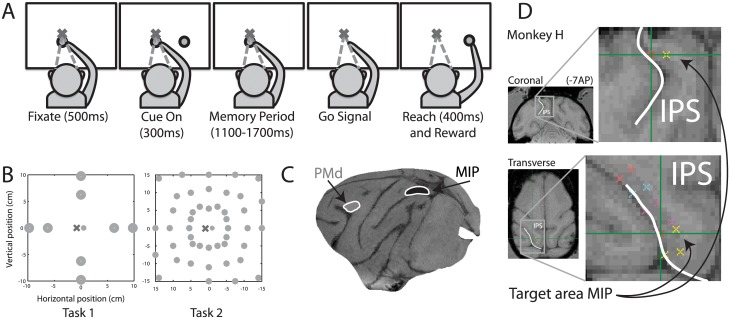
**(A)** Variable amplitude memory reach task. To initiate a trial, monkeys had to touch a central green circular target and visually fixate a red target located in the center of a touch screen for 500ms (fixation). A cue (circular green target) was then flashed in the periphery for 300ms indicating the target of the upcoming reach. A randomized delay period followed (memory period: 1.1–1.7 s) during which monkeys had to maintain the visual fixation and the hand contact established in the fixation period. The central green target was then extinguished instructing the monkeys to reach to the location indicated by the cue flash. Monkeys had to touch the screen within a 2.5 cm circle centered on the target for 400ms without breaking visual fixation. If successful, monkeys were rewarded with juice. **(B)** Target configurations. Monkeys reached to targets in two different configurations. In Task 1, targets were arranged at two amplitudes along each of four equally distributed directions. In Task 2, targets were at three amplitudes in each of 16 equally spaced directions, slightly offset from a perfectly radial configuration to maximally use the touchscreen area. **(C)** Electrophysiological recording anatomy. The approximate anatomical location of chronic microelectrode array implantation is shown for MIP and PMd. (**D**) Structural MRI with surgery plan targeting area MIP in one monkey. The intra-parietal sulcus (IPS) is marked for anatomical reference. The cross-hairs indicate the center of the targeted location, and the ticks represent the boundary of the cortical surface of interest.

Data was collected from three awake, behaving male rhesus monkeys (Macaca Mulatta, F, H, S), weighing between 5.6 Kg and 11.9 Kg. Monkeys were first implanted with a MRI-compatible head post (Rogue Research) and trained on the experimental paradigm until they were thoroughly familiar with the task. We then performed a second surgery to implant a 2-cm circular recording chamber (Crist Instrument, IAC series) on the contralateral side of the reaching right arm of *monkeys H* and *F*. Chamber placement was guided stereotaxically and centered at posterior 7 mm, lateral 5 mm in *monkey F*. For *monkey H*, we placed the chamber in a position that maximized access to the medial bank of the intraparietal sulcus as confirmed by Brainsight, a neuro-navigation system (Rogue Research); surgery plan images centered on the targeted area are shown in Fig 1D. We secured the chamber in place with MRI-compatible ceramic screws (Rogue Research) and Simplex P bone cement (Stryker, Hamilton, ON, Canada). Monkey S was implanted with two 16-channel micro-electrode arrays (MicroProbe, Gaithersburg, MD) each in areas MIP and PMd of the left hemisphere (64 channels in total). Micro-electrode array locations were planned using Brainsight, by first registering sulcal markers (the intraparietal sulcus) to a stereotaxic atlas registered to the animal’s structural MRI. During surgery, a motion-capture system using skull-referenced fiducial points was used to precisely locate the planned array implantation site (Rogue Research). In both acute and chronic recording setups, we recorded from deep within the sulcus (2-8mm for single electrode recordings, 3-6mm for micro-electrode arrays) to exclusively target area MIP. The electrode depth measurements were made using a stereotaxic atlas registered to each animal’s structural MRI. The approximate anatomical location of chronic microelectrode array implantation is shown in Fig 1C. Additional surgical and experimental procedure details are reported elsewhere [29]. All surgical and experimental procedures complied with the Canadian Council of Animal Care guidelines and were approved by the McGill Animal Care Committee. Monkeys were weighed and their health and growth monitored daily. Animals were also pair-housed and had constant access to exercise via a jungle gym on all days, including experimental days. Each monkey had its own recording schedule that ensured that experiments started at the same time every session. Monkeys were given fresh fruits in the lab after all completed sessions.

The monkeys performed the experiment in a dimly lit Faraday chamber. Each animal was seated in a chair within arm’s reach (~45cm) of a touch screen (ELO Touch, California) that was coupled to a monitor. The only light in the chamber emanated from visual cues displayed on the monitor and the monitor’s low ambient output. Monkeys reached the touch screen through an opening in the chair on the side of their reaching hand (right arm in all animals). The non-reaching hand was obstructed. The monitor and touchscreen were on the fronto-parallel plane, within arm’s reach of the animal. Behavior was controlled by a real time system (LabVIEW RT, National Instruments). Eye position was monitored with an infrared reflection camera (ISCAN, Boston) and hand position was monitored using the acoustic touch screen.

The single unit recording procedure is reported in detail elsewhere [29]. Briefly, extracellular recordings in monkeys *F* and *H* were performed with a single channel from a multichannel micromanipulator system (NAN Drive, NAN Instruments). Before each recording session, a 23-gauge stainless steel guide tube containing a 120-mm tungsten microelectrode (FHC, Pl/Ir impedance: 1 MΩ) was placed over the appropriate cortical location (in the *XY* plane) and lowered onto the dura mater surface. Thus the guide tube served as the electrical ground. The electrode was then lowered into the cortex to a desired depth ranging from 2 to 8 mm and left to rest from 15 to 60 min before any subsequent movements. All neurons that could be isolated and stabilized were recorded. Single-/multi-channel recordings were filtered (250Hz– 8000 Hz for spikes) and amplified using commercially available systems (Plexon Inc, Texas). Each channel was digitized (at sampling rates of 40 kHz for spikes) and continuously recorded to disk for further analysis. Spike waveforms were sorted online using a manual-sorting method (Plexon Inc.), and offline using custom software (MATLAB) before being visually inspected.

### Behavioral task and datasets

We trained the monkeys on the memory reach task, illustrated in Fig 1A, for several months before collecting data. To initiate a trial, monkeys had to touch a central green circular target and visually fixate a red target located in the center of a touch screen for 500ms (fixation). A cue (circular green target) was then flashed in the periphery for 300ms indicating the target of the upcoming reach. A randomized delay period followed (memory period: 1.1–1.7 s, uniformly distributed) during which monkeys had to maintain the visual fixation and hand contact established in the fixation period. The central green target was then extinguished, instructing the monkeys to reach to the location indicated by the cue flash. Monkeys had to touch the screen within a 2.5 cm circle centered on the target for 400ms without breaking visual fixation. If successful, monkeys were rewarded with juice.

We used two different reach target configurations (see Fig 1B): Task 1 consisted of targets at two amplitudes along each of four equally distributed directions, while Task 2 consisted of targets at three amplitudes in each of 16 equally spaced directions. The reach targets subtended the near periphery (±12.5 degrees of visual angle for Task 1 and ±25 degrees of visual angle for Task 2). Reach targets in Task 2 were slightly offset from a perfectly radial configuration to maximally use the touchscreen area (see Fig 1B right panel). We first recorded 93 MIP neurons while monkeys F and H reached to targets in Task 1. Using chronically implanted micro-electrode arrays, we recorded 39 MIP neurons and 42 PMd neurons from monkey S performing Task 1. In order to characterize the spatial response patterns of neurons, we recorded an additional dataset of 58 MIP neurons and 52 PMD neurons in one session of monkey S reaching to targets in Task 2 (see Fig 1B). Three out of these 48 targets were omitted due to insufficient number of trials. In total, we collected 42 (30) trials per target for Task 1, and 10 (1) trials per target for Task 2 (median and interquartile range).

In total, 190 MIP neurons (33, 60, and 97 from monkeys F, H, and S respectively) and 94 PMd neurons (from monkey S) were recorded. We analyzed the firing rates of these neurons in four different trial epochs (cue, early-memory, late-memory, movement) of length 500ms. We defined the criterion for task-relatedness as significantly different spiking activity during at least one of these four trial epochs relative to a baseline epoch (p < 0.05/4, t-test with Bonferroni correction for multiple comparisons); importantly, this criterion for task-related neurons is not based on tuning properties such as selectivity for amplitude or direction. Based on this task-relatedness criterion and a threshold on firing rates (>1Hz in at least one of the four trial epochs considered), 152 MIP neurons and 68 PMd neurons were selected for analysis in the current study. Data and analyses are made available on a public repository: https://github.com/RishiRajalingham/musallam_plos2017.

### Analysis

We computed firing rates in four trial different epochs, of 500ms bins aligned to trial events: Cue (C): [0ms, 500ms] after target cue onset; Early-Memory (EM): [200ms, 700ms] after target cue offset; Late-Memory (LM): [-700ms, -200ms] before go signal; Reaction and Motion Time (RT/MT): [0ms, 500ms] after go signal. The early and late memory period epochs (EM and LM) were temporally offset from cue offsets to avoid contamination by visual and preparatory movement signals respectively. As such, the early and late memory period epochs could overlap slightly, with an average overlap of 0ms. The RT/MT epoch overlapped substantially with the reaching movement, as the monkeys’ reaction times (time from go signal to start of hand movement) and motion times (time from start of hand movement to target acquisition) were 276ms (±29ms) and 186ms (±38ms) respectively. As such, the reach and gaze positions were spatially dissociated during this epoch.

### Single unit analyses

We examined the modulation of neuronal firing rate by the instructed reach amplitude and direction. To assess significant modulation in firing rate due to these task variables, we used multivariate (2-way) fixed-effect ANOVAs with interaction of firing rate with direction and amplitude as factors. ANOVAs were approximately balanced, as the factors (reach target direction and amplitude) were sampled from a uniform distribution. To further assess significant modulation by amplitude in each direction separately, we used unpaired t-tests between firing rate distributions in small and large amplitude trials. When testing for significant effects in at least one of the four epochs of interest, we adjusted the significance threshold (Bonferroni correction) to counter problems of multiple comparisons. When testing significance of a one-group proportion against chance (e.g. proportion of neurons with significant amplitude modulation per area), we used a one-tailed binomial test. We use the convention of denoting p < 0.05 as *, p < 0.01 as ** and p < 0.001 as *** throughout.

### Response field characterization

In order to characterize the spatial response fields of neurons, we examined the firing rate of neurons recorded as monkey S reached to 3 amplitudes in each of 16 directions. We first mapped targets to Cartesian coordinates and used linear interpolation to estimate the firing response at each point in a Cartesian grid (11x11 bins, spanning 14cm x 14cm). The interpolation procedure simply estimates neuronal responses for arbitrary target locations within the experimentally measured reach target space, using a spatial smoothness prior on firing rates. We quantitatively characterized the interpolated response patterns by measuring the eccentricity of the peak response coordinate. Additionally, we measured the response field width, defined as the square root of the spatial area containing the top quartile of responses. Both response field eccentricity and width were converted to degrees of visual angle, based on a 45cm eye-to-screen distance.

### Response field modeling

**Fig 2:**
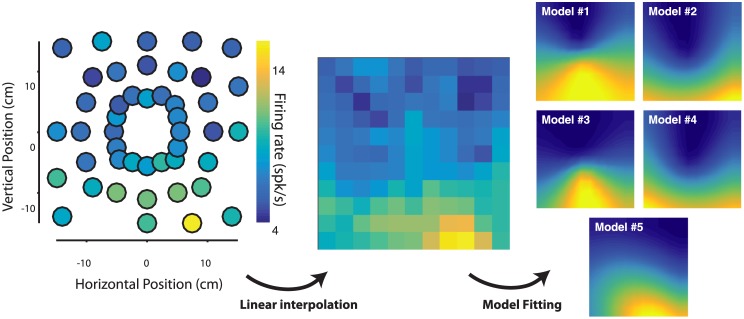
Response field characterization and modeling. For an example PMd neuron, the response field characterization procedure is shown. Color scale indicates firing rate response. The mean firing rate response to each reach target (left panel) is used to interpolate the two-dimensional spatial response pattern (middle panel). This interpolated response pattern is then used to fit computational encoding models of neuronal responses. Model response patterns for each of the tested models (corresponding to models 1–5) are shown below.

To quantitatively test several alternative hypotheses of neuronal encoding, we constructed and tested the goodness of fit of several computational models (see Table 1, Fig 2). Our primary goal was to uncover models of neuronal response patterns with good generalization accuracy, i.e. models that accurately capture the data without over-fitting it, such that they can generalize to new, unseen data. To this end, we used cross-validated accuracy, wherein a fixed learned model is explicitly tested on held-out data [30]. This metric directly measure a model’s generalization ability rather than estimate it indirectly based on model parsimony, as is the case with measures such as Akaike’s information criterion (AIC) or Bayesian information criterion (BIC) [30]. Moreover, AIC and BIC metrics do not apply in a cross-validation scheme, as all cross-validated models are fixed (have zero free parameters) with respect to the test data [30]. The models and the procedure used to estimate their goodness of fit with the neuronal data are described in detail below, fully characterized in Table 1, and illustrated conceptually in Fig 2.

**Table 1:** Description of all computational models tested, with mathematical characterizations, explicitly stated optimization parameters and constraints.

Model	Equation	# Params	Constraints	Description
1	*f*(***x***) = cos(*θ* − *θ*_0_)	1	*θ*_0_ ∈ [−*π*, *π*]	Cosine directional tuning
2	*f*(***x***) = cos(*θ* − *θ*_0_) * *kr*	2	*θ*_0_ ∈ [−*π*, *π*], *k* ∈ [−100,100]	Cosine directional tuning with linear amplitude gain
3	$f\left(x\right)=\frac{e^{\sigma \text{cos}\left(\theta -\theta_{0}\right)}}{2\pi I_{0}\left(\sigma \right)}$	2	*θ*_0_ ∈ [−*π*, *π*], *σ* ∈ [0.01, ∞]	Von Mises directional tuning
4	$f\left(x\right)=\frac{e^{\sigma \text{cos}\left(\theta -\theta_{0}\right)}}{2\pi I_{0}\left(\sigma \right)}*\;kr$	3	*θ*_0_ ∈ [−*π*, *π*], *σ* ∈ [0.01, ∞], *k* ∈ [−100,100]	Von Mises directional tuning with linear amplitude gain
5	$f\left(x\right)=e^{{\left(\text{x}-\mu \right)}^{T}\Sigma^{-1}\left(\text{x}-\mu \right)}$,***μ*** = [*μ*_*x*_ *μ*_*y*_], $\Sigma =\left[\begin{array}{cc} \sigma_{x}\sigma_{x} & \rho \sigma_{x}\sigma_{y}\\ \rho \sigma_{x}\sigma_{y} & \sigma_{y}\sigma_{y} \end{array}\right]$	5	*μ*_*x*_, *μ*_*y*_ ∈ [−20, 20], *σ*_*x*_, *σ*_*y*_ ∈ [−5, 50]	Gaussian position tuning

For each neuron and each epoch, we first split the trials into two halves. From these two disjoint sets of trials, we estimated two response patterns N1 and N2 using linear interpolation as described above. The interpolated response patterns were mid-range normalized ($FR\leftarrow \frac{FR-\text{min}\left(FR\right)}{\text{max}\left(FR\right)-\text{min}\left(FR\right)}\frac{}{}$) to map onto [0,1]. The Pearson correlation between N1 and N2 is the *neuronal internal consistency*, a measure of the reliability of the neuronal response pattern estimated across disjoint sets of observations. This value corresponds to a noise ceiling of the neuronal data, as it is measured relative to the neuron’s best possible model, namely itself. From each response pattern, we solved for the model parameters with constrained non-linear least-squares minimization (see Table 1 for parameters and parameter constraints), resulting in two model response patterns M1 and M2. The Pearson correlation between N1 and M2 (and between N2 and M1) is the cross-validated correlation (*ϱ*_*neuron*,*model*_), as it compares the neuronal response pattern to the prediction from a model fitted on different sets of observations (trials). This cross-validation procedure is crucial to ensure that model fits do not reflect over-fitting. For each neuron and each epoch, we repeated this procedure 50 times with random resampling of trial split-halves to estimate average values for correlation values. For brevity, we refer to the cross-validated correlation between model and neuronal response patterns as the *model consistency*. Using this method, we tested the consistency of several models that each map a two-dimensional point ***x*** = (*x*, *y*) = (*θ*, *r*) to a scalar normalized firing rate value *FR*(**x**). As with the neuronal response patterns, the model response patterns were mid-range normalized ($FR\leftarrow \frac{FR-\text{min}\left(FR\right)}{\text{max}\left(FR\right)-\text{min}\left(FR\right)}$) to map onto [0,1].

All five tested models are listed in Table 1 and visualized for an example neuron in Fig 2. We tested for directional tuning using both cosine and Von Mises functions, parameterized by preferred direction angle and width (concentration parameter). For each of these, we tested either a pure directional tuning, or directional tuning with a linear amplitude gain (parameterized by a gain *k*). The resulting four model equations are shown in Table 1 (Models 1–4). We also tested a model of position tuning (see Model 5 in Table 1), consisting of a 2D Gaussian function, characterized by parameters ***μ*** and ***Σ*** denoting the two-dimensional center and width of the Gaussian respectively. This model corresponds to a smooth response field that fires maximally at a particular location in reach space. Example response patterns for each of these models is shown in Fig 2.

We modeled the response patterns of all task-related neurons recorded under Task 2, separately for each epoch. As noted above, the models were fitted and/or optimized using only the training data, and subsequently tested on held-out testing data to measure model consistency. Importantly, this cross-validation procedure avoids the problem of over-fitting, (where excessively complex models, i.e. models with large number of parameters, fit the noise in the data). By always measuring model consistency on held-out data, we ensure that differences in consistencies across models are not due to their ability to over-fit (i.e. number of parameters).

## Results

**Fig 3:**
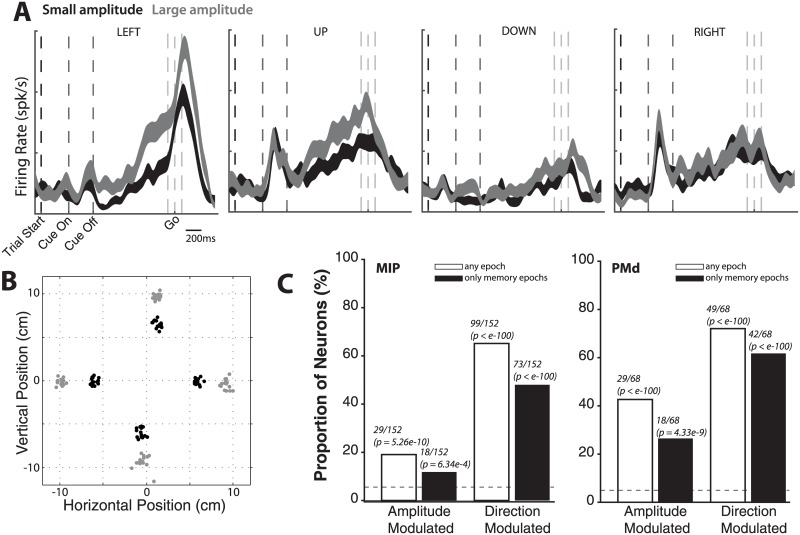
**(A)** Example neuronal data. For an example neuron recorded under Task 1, the spike density functions (mean ± SE) for small and large amplitude is shown (small and large amplitude are shown in grey and black respectively). Each panel corresponds to a reach direction. Vertical lines mark trial events; the go signal is shown as mean +- SE due to the randomized length of the memory period (see dashed line). Axes are identical for the four panels. For this neuron, we observe significant modulation of memory period spiking activity by reach amplitude in some but not all directions. **(B)** Example behavioral data from one session. The endpoint of reaches to target in Task 1 are shown. For visual clarity, 15 trials for each target are shown. Small and large amplitude are shown in grey and black respectively. **(C)** Population analysis of modulation by reach direction and amplitude for both tasks. The proportions of task-related MIP and PMd neurons with significant main or interaction effects of reach amplitude (and reach direction), in any of the four trial effects (p < 0.05/8 with Bonferroni correction for eight comparisons across effect types and epochs; 2-way ANOVA interaction model) are shown (white bars), along with the “null” proportion expected by chance (dashed line). The corresponding proportions when considering only movement planning epochs (p < 0.05/4, Bonferroni correction for four comparisons), are also shown (black bars).

Our primary goal was to test the hypothesis that the amplitude of a planned reach is represented in the activity of neuronal populations in MIP. We also set out to compare any amplitude encoding in MIP to the corresponding representation in PMd. To do so, we recorded single neuron activity from monkeys (three monkeys for MIP, one monkey for PMd) performing a variable amplitude memory reach task (see Materials and methods). The task, as illustrated in Fig 1A, required the monkeys to accurately reach to a remembered visual target. Reach targets were arranged in two different configurations (see Fig 1B): Task 1 consisted of targets at two amplitudes along each of four equally spaced directions, while Task 2 consisted of targets at three amplitudes in each of 16 equally spaced directions. Fig 3A and 3B show example neuronal and behavioral data for an MIP neuron recorded under Task 1. For visual clarity, the behavioral data (Fig 3B) only show the endpoint of 15 reaches per target (small, large amplitude shown in black, grey respectively). The four panels in Fig 3A show the example neuron’s spike density functions (mean ± SE) for small and large amplitude, separately for each reach direction. For this neuron, we observe significant modulation of memory period spiking activity by reach amplitude in some (UP, LEFT) but not all directions, suggesting that neuronal responses are modulated by reach amplitude, possibly as an interaction of reach direction and reach amplitude.

To quantify this phenomenon at the population level, we measured the frequency of occurrence of significant main and interaction effects of reach amplitude and reach direction (2-way ANOVA interaction model) in each of four trial epochs, for populations of neurons recorded from MIP and PMd under both target configurations. We restricted this analysis to the subset of “task-related” neurons, defined as cells with significantly different spiking activity during at least one trial epoch (Cue (C), Early Memory (EM), Late Memory (LM), Movement (RT/MT)) relative to the baseline pre-trial epoch (p < 0.05/4, t-test with Bonferroni correction for multiple comparisons). Importantly, this criterion for task-related neurons is not based on tuning properties such as selectivity for amplitude or direction, and thus does not overestimate the measured proportions of neurons.

We first counted the number of neurons with significant main or interaction effects of reach amplitude, in any of the four trial epochs (p < 0.05/8 with Bonferroni correction for eight comparisons across effect types and epochs; 2-way ANOVA interaction model). Fig 3C shows the proportion of task-related MIP and PMd neurons that satisfied this criterion (see white bars). The “null” proportion expected by chance (dashed line, obtained by performing the same analysis on randomly shuffled data 100 times) is shown for comparison. This proportion was statistically significant (MIP: 19%, p = 5.26e-10; PMd: 43%, p < e-100; binomial test against 5% null). Furthermore, this result holds even when restricting to only movement planning epochs (p < 0.05/4, Bonferroni correction for four comparisons), albeit with smaller proportions of neurons (MIP: 12%, p = 6.34e-4; PMd: 26%, p = 4.33e-9; binomial test against 5% null). Thus, a small but statistically significant proportion of task-related neurons in both MIP and PMd exhibit spiking responses modulated by reach amplitude.

**Fig 4:**
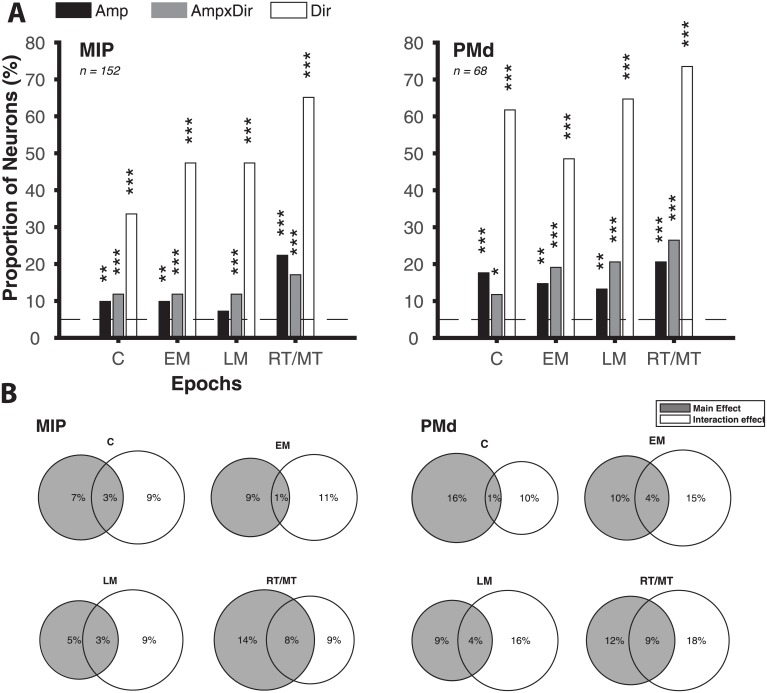
**(A)** Population analysis of modulation by reach direction and amplitude for both tasks. The proportions of neurons that varied their activity significantly due to the instructed reach direction (black bars), instructed reach amplitude (gray bars), and an interaction of direction and amplitude (white bars) are shown for each of four trial epochs. The two panels correspond to task-related neurons recorded from MIP and PMd respectively, pooling across both target configurations. The proportion expected by chance (“null”, obtained by randomly shuffling the data) is shown with the black horizontal line. Asterisks indicate significance of proportions relative to chance (*: p < 0.05, **: p < 0.01, ***: p < 0.001). **(B)** Main and interaction effects of reach amplitude. For each cortical area and trial epoch, a Venn diagram shows the proportion of task-related neurons with a significant main effect (gray discs) and interaction effect (white discs) of reach amplitude. The overlap in the two discs (light gray) indicate the proportion of neurons exhibiting both significant main and significant interaction effects due to reach amplitude.

For task related neurons, we examined the dependence of amplitude sensitivity on the trial epoch (cue, early memory, late memory, and motion epochs) and effect type (main versus interaction effect). Fig 4A shows the proportion of task-related MIP and PMd neurons with responses significantly modulated by main and interaction effects of reach amplitude, for each of four trial epochs (main effect: gray bars; interaction effect: white bars; p < 0.05, 2-way ANOVA interaction model). Again, the corresponding proportions of task-related neurons with significant main effects for reach direction (white bars), as well as the “null” proportion expected by chance (dashed line, obtained by randomly shuffling the data 100 times), are shown for comparison. Fig 4B shows these proportions of neurons with significant main and interaction effects for each epoch separately, including the overlap in populations selected from these criteria (see Venn diagrams).

In particular, the prevalence of interaction effects suggest a joint encoding of direction and amplitude by neurons in both areas. To examine the dependence of amplitude selectivity on reach direction, we performed post-hoc tests measuring the number of reach directions in which task-related neurons showed significant modulation due to reach amplitude. For each neuron recorded under Task 1 (two amplitudes in each of four directions), trials were separated by reach direction and t-tests were used to infer significant differences between responses of small and large amplitudes, for each direction. We found that over one fifth of task-related MIP neurons exhibited amplitude selectivity in exactly one out of the four directions for all epochs (C: 22%, EM: 18%, LM: 18%, RT/MT: 28%), while a smaller proportion were selective to reach amplitude in two directions (C: 1%, EM: 6%, LM: 4%, RT/MT: 4%). Less than 1% of task-related MIP neurons showed selectivity to reach amplitude in more than two directions. PMd exhibited similar patterns of amplitude selectivity; approximately a third of all neurons were selective to amplitude in one direction: (C: 29%, EM: 21%, LM: 36%, RT/MT: 36%), but this proportion decreased significantly for selectivity in two directions (C: 0%, EM: 4%, LM: 7%, RT/MT: 7%), with zero neurons showing amplitude selectivity in more than two directions in any epoch. Thus, all task-related neurons, in both MIP and PMd, show significant modulation of firing activity by reach amplitude in at most some but not all directions.

**Fig 5:**
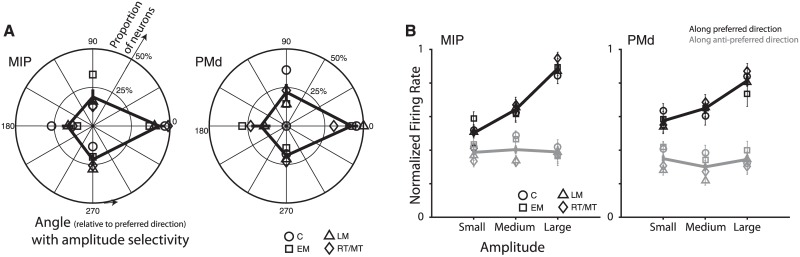
**(A)** Bias in directions with amplitude-selectivity. Each polar histogram shows the proportion of neurons as a function of ΔPD, the difference in angle between directions with significant amplitude modulation and the preferred direction of the neuron. The two panels correspond to neurons recorded from MIP and PMd under Task 1; different markers correspond to the four epochs of interest. Significant amplitude modulation is tuned towards the preferred direction of the neuron. **(B)** Bias in amplitude response. Each subpanel shows normalized firing rate response to reaches to small, medium and large amplitude targets of all task-related cells recorded under Task 2. Markers indicate responses for each epoch, and solid lines indicate the pooled average (mean ± SE over neurons shown). Data are separated by responses along the preferred (black line) and anti-preferred (gray line) directions.

Furthermore, directions with significant modulation by amplitude often coincided with the preferred direction of the neuron. For each neuron, we defined the preferred direction as that which resulted in maximum mean firing rate, for each epoch separately. For each epoch, we then computed ΔPD, the difference in direction angle between this preferred direction and the direction with “greatest amplitude modulation,” i.e. the direction with minimal p-value for amplitude modulation, from unpaired t-tests. This analysis only included neurons with significant amplitude modulation in at least one direction (p < 0.05, unpaired t-tests for each direction) recorded under Task 1. Fig 5A shows the proportion of these amplitude selective neurons in MIP and PMd as a function of the ΔPD, for each epoch; the data pooling over all epochs is shown in black. We observe that significant amplitude modulation is tuned towards the preferred direction of the neuron in both MIP and PMd. The histogram of ΔPD was significantly non-uniform (MIP: p = 6.79e-5; PMd: p = 0.03; Rayleigh test for circular uniformity) and centered near 0° (MIP: -6.71° ± 6.19°; PMd: 4.76° ± 10.34°; circular mean ± SE). This result holds when ΔPD is computed relative to the circular average of all directions with significant amplitude modulation, rather than the direction with greatest amplitude modulation (MIP: p = 3.6e-4, centered at -2.07° ± 6.30°; PMd: p = 0.01, centered at -12.61° ± 10.07°).

In particular, neuronal responses in both areas most consistently peaked for reach targets at large amplitudes along the preferred direction. Fig 5B shows the normalized firing rate response to reaches to small, medium and large amplitude targets along the preferred and anti-preferred directions of all task-related cells recorded under Task 2 (mean ± SE over neurons shown, for each epoch). We observe that the largest amplitude target along the preferred direction consistently resulted in the greatest neuronal response. Pooling over all epochs, there is a significant positive correlation between the target amplitude and the normalized neuronal firing rate along the preferred direction (MIP: *r = 0*.*46*, *p = 7*.*25e-28*; PMd: *r = 0*.*29*, *p = 1*.*83e-10*), but not along the anti-preferred direction (MIP: *r = 0*.*01*, *p = 0*.*84*; PMd: *r = -0*.*02*, *p = 0*.*66*). In brief, neurons in MIP and PMd exhibit evidence for a joint encoding of direction and amplitude, wherein modulation by amplitude is most likely to be observed and strongest along the preferred direction of the neuron, with maximal response to more peripheral targets along the preferred direction.

### Neuronal spatial response patterns

**Fig 6:**
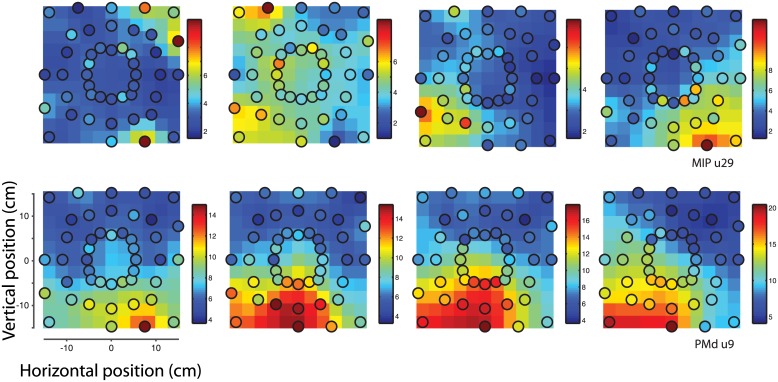
Example spatial response patterns. Each row shows the spatial response patterns of an example neuron, for each epoch considered. One example neuron from each area is shown. The measured average firing rates at spatial positions of the reach target are plotted (colored circles) overlaid on the interpolated spatial response pattern (11x11 colored maps), using the same color scale. Warmer colors indicate higher firing rates. For visualization purposes, the interpolated spatial response patterns have been slightly smoothed, using a two-dimensional Gaussian kernel of width 0.5 bins.

Together, these results indicate that the direction and amplitude of a reach target in space conjointly modulate neuronal activity in MIP and PMd. The interaction between amplitude and direction suggests a possible encoding of the position of a target as a coordinate in visual space, rather than a representation of direction and/or amplitude. These different encoding hypotheses are not optimally differentiated using the sparse reach target configuration in Task 1. In order to do so, we characterized the response patterns of task-related neurons in MIP and PMD simultaneously recorded while one monkey performed reaches to a dense spatial sampling of targets (16 directions x 3 amplitudes, referred to as Task 2). We analyzed the spatial response patterns of neurons using the procedure (described in Methods) illustrated in Fig 2. Briefly, the average firing rates of neurons in response to reaches to the different targets were used to interpolate and characterize their spatial response patterns in a 2D fronto-parallel plane, for each neuron and each epoch. To test our encoding hypotheses, we compared the cross-validated goodness of fit (consistency) of several alternative computational models with respect to the interpolated response patterns of single neurons. Fig 6 shows spatial response patterns for two example neurons, one from each area, for all epochs considered. The measured average firing rates at spatial positions of the reach target are shown (colored circles) overlaid on the interpolated spatial response pattern. We first qualitatively observe that neurons exhibit reliable spatially tuned responses in both areas. Further, these example response patterns suggest that neurons are not simply modulated by target direction alone, but rather seem to encode particular regions of reach-space in each epoch. As previously shown (see Fig 5B), response fields typically peaked in the periphery. Quantitatively, the eccentricities of the peak response locations were not different between MIP and PMd (MIP: 16.04° ± 0.43°; PMd: 16.05° ± 0.44°, mean ± SE, pooling across epochs). Response field widths were slightly but significantly larger in MIP than in PMd (MIP: 11.28° ± 0.07°; PMd: 10.89° ± 0.09°, p < 1e-4, unpaired t-test, see Methods). We additionally observe that the spatial response patterns of neurons can vary across epochs. However, these temporal dynamics were considered beyond the scope of the current study.

To quantitatively test alternative encoding hypotheses of neuronal responses, we asked how well several alternative computational encoding models could predict the observed neuronal responses. The tested encoding models, ranging from cosine directional tuning to Gaussian position encoding, are mathematically described in Table 1 and illustrated with example model fits in Fig 2. The large number of targets in Task 2, and corresponding small number of trials per target condition, could result in a relatively noisy estimate of average neuronal firing rate for each target. We measured this noise ceiling via the neuronal internal consistency (the correlation between neuronal response patterns estimated from disjoint split halves of the data). Importantly, the entire modeling procedure was cross-validated: the model training and testing were done on separate disjoint halves of the data (see Methods for detailed description of modeling procedure). Cross-validation ensures that differences in consistencies across models are not due to their ability to over-fit (i.e. are not due to differences in number of parameters).

**Fig 7:**
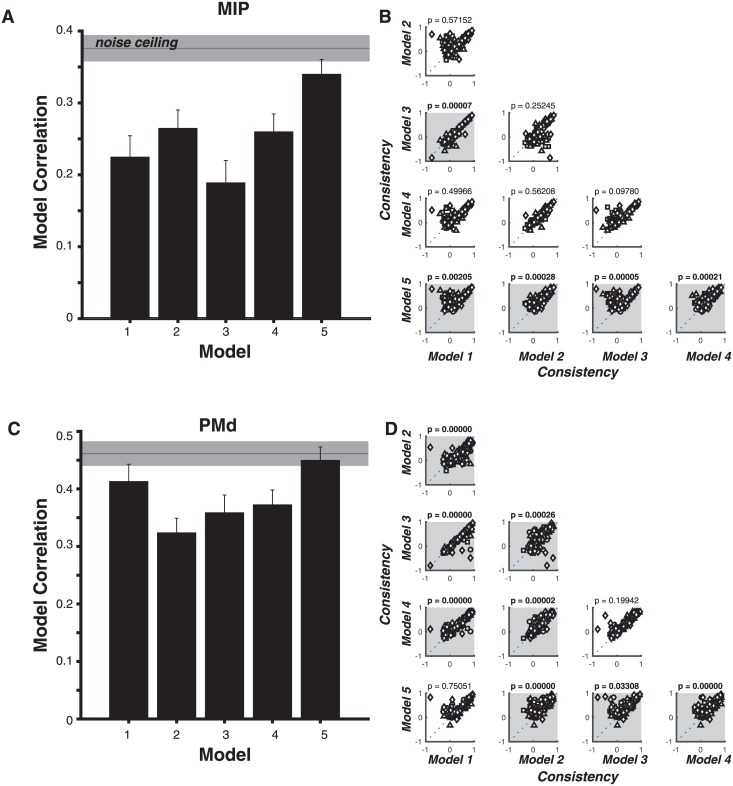
**(A)** Model Consistency. For each of the five tested models, the resulting distribution of model consistencies over all task-related MIP neurons and all epochs (mean ± SE) is shown for each model. The gray horizontal bar corresponds to the noise ceiling (internal consistency of the neuronal response patterns, mean ± SE). **(B)** Quantitative comparison for each pairwise combination of models. Each panel (row i, column j) directly compares two models (model #i vs. model #j), and each point in the panel’s scatter plot corresponds to a particular neuronal response pattern in MIP. The dashed unity line corresponds to equal consistency across these two models. The p-value obtained from a two-tailed signed rank test comparing the distributions of correlations between models *i* and *j* is shown in the panel title, and model pairs with statistically significant differences are highlighted in grey. **(C, D)** Identical to (A,B) for task-related neurons in PMd.

For each of the five tested models, we computed a consistency value for each task-related neuron recorded under Task 2 and for each epoch. Fig 7A shows the resulting distribution of model consistencies over neuronal samples (mean ± SE) for each model. First, we observe that all models capture a relatively large proportion of the replicable variance in neuronal responses (i.e. the model consistencies are relatively close to the noise ceiling of the data). However, we observe a significant improvement in model consistency for the Gaussian position encoding model (model 5), relative to null models of direction and amplitude tuning (models 1–4). Fig 7B shows this quantitative comparison for each pairwise combination of models. Each panel (row i, column j) directly compares two models (model #i vs. model #j), and each point in the panel’s scatter plot corresponds to the models’ consistency to a particular neuronal response pattern. The dashed unity line corresponds to equal consistency across these two models. The p-value obtained from a two-tailed signed rank test comparing the distributions of correlations between models *i* and *j* is shown in the panel title, and model pairs with statistically significant differences are highlighted in grey. We observe that the Gaussian position-encoding model (#5) significantly outperforms all other models. In particular, these comparisons suggest that for a subset of neurons (points along the identity line), the consistencies of direction and position encoding models are largely indistinguishable, whereas for a sizeable proportion of neurons, the position-encoding model (model #5) vastly out-performs the direction-encoding model (see points above the unity line). Taken together, these results suggest that a simple Gaussian position-encoding model most accurately captures the response patterns of task-related neurons in MIP.

Fig 7C and 7D repeat this procedure for task-related PMd neurons, with very similar results. However, both cosine direction-encoding (model 1) and Gaussian position-encoding (model 5) accurately capture the neuronal response patterns, significantly better than all other models, but these data do not have the power to distinguish between these two models.

## Discussion

### Representation of reach amplitude and direction in MIP

In this work, we tested the hypothesis that the reach amplitude is represented in the activity of neurons, during movement planning and execution, in sensory-motor integration area MIP, a sub-region of the parietal reach region. Previous work has examined the neuronal representation of the in-plane depth of reaches in neighboring and overlapping reach-related posterior parietal areas [11–13, 31]. Here, we found that neurons in MIP, similarly to PMd, conjointly represent the intended reach direction and amplitude, in a manner consistent with encoding of the remembered location of the reach goal.

Similar to previous findings in motor and premotor cortices [4, 20], our results highlight an under-representation of reach amplitude relative to reach direction in movement planning signals; the number of neurons with significant modulation by reach amplitude is approximately one third of the corresponding number for reach direction. Such a comparison may not be valid given that the two variables were not matched in dynamic range: while the full range of reach directions in the fronto-parallel plane were presented, the tested reach targets extended a limited range of amplitudes. However, the observed differences in representation of direction and amplitude are reflected in the reaching behavior. Psychophysical experiments in humans show that subjects make greater errors in the extent of a reach compared to the direction [32], and that the initial kinematics of a movement do not perfectly predict movement amplitude without the inclusion of compensatory adjustments [33]. These behavioral data point towards a poorer representation of reach amplitude, relative to direction, in the movement plan. However, reach amplitude must be represented in the neuronal motor plan, because the visual stimuli defining the reach goal no longer exist in the external environment in the context of memory-guided reaches. As such, the modest neuronal modulation of movement planning signals by amplitude observed both here and in previous studies is consistent with the behavioral output.

We focused our analyses on neuronal activity in MIP (152 task-related neurons in monkeys *H*, *F*, *S*), and recorded from PMd (68 task-related neurons in monkey *S*) for direct cross-area comparisons. We did not observe any idiosyncratic behavioral or neuronal signatures in monkey S and, importantly, multi-electrode recordings in monkey S simultaneously sampled from both MIP and PMd in each session, thus eliminating the possibility of experimental variance that selectively affects one brain area, and maximizing power for direct comparisons. Furthermore, our recordings in PMd largely replicate previously reported findings characterizing this area, namely that the activity of PMd neurons is modulated by an interaction of reach direction and amplitude [20], and likely to represent the instructed target location [34]. Thus, we argue that this data has sufficient power to support inferences about the representation of reach goal amplitude in MIP, as compared to PMd.

Neurophysiological recording sites were targeted for area MIP based on a stereotaxic definition. Recording chambers for acute recordings were placed sufficiently posterior (-7) such that area 5 is restricted to the gyral surface, and our electrode penetrations were deep enough (2-8mm) to exclusively target putative area MIP. For chronic array recordings, we used long and staggered electrodes (3-6mm), thus restricting the recording sites to deep within the sulcus at the planned location. Using a neuro-navigation system (Brainsight), we were able to precisely register the stereotaxic atlas to individual subject structural MRI scans (for monkey H, S) for targeting and surgery planning. However, the boundary between MIP and neighboring cortical area 5 is not stereotaxically well defined, and the denominations are not always consistent across studies [35]. In this study, we did not perform histological validation of recording site locations, which may help differentiate these two neighboring areas [36], but rather rely on stereotaxic planning to claim that recordings targeted area MIP.

We trained monkeys to perform memory-guided reaches in order to investigate the representation of reach direction and amplitude in movement planning signals, independently of visual responses. We observed that monkeys accurately reached to the remembered target, with an average (± SEM) error of 1.91° (± 0.05°); this suggests that closed-loop visual feedback, while likely extremely useful [37–39], may not be necessary for making correct reaching movements, especially in the domain of highly trained movements. We found that a modest but significant proportion of neurons in MIP and PMd modulate their movement planning activity based on both the direction and the amplitude of the forthcoming reach, suggesting that these neuronal representations may causally support accurate memory-guided reaching behavior. Furthermore, we speculate that these same representations may contribute to planning and executing visually-guided reaches [40].

We examined the representation of reach direction and amplitude at various epochs within a memory reach trial. It is important to note that the cue epoch (C) overlaps with the visual stimulus, whereas the movement epoch (RT/MT) overlaps with a hand movement. The observed selectivity to reach target direction and amplitude during the former (C) could reflect visual response selectivity. Likewise, the selectivity during the latter (RT/MT) could reflect a representation of the hand position. Moreover, since we did not vary eye position (monkeys held gaze fixation throughout the trial), these data do not provide evidence for or against an eye-centered representation, i.e. a representation of the reach goal or hand position relative to the center of gaze.

Finally, we found a bias in the representation of reach space in our recorded neuron population, with systematically stronger response for planned reaches to large amplitudes (see Fig 4). Importantly, this response bias does not necessarily suggest that the periphery is better represented; indeed, the high-dimensional neuronal population can accurately represent both central and peripheral reach targets without having a substantial proportion of individual units respond with high firing rates to these targets. Additionally, this finding may depend on our choice of reach targets positions, where the observed peak locations correspond to ceiling effects from insufficient spatial sampling. However, in this work, we used reach targets with eccentricities at near practical limits (to subtend the entire touchscreen surface for Task 2), comparable or greater in extent relative to previous similar work [20, 41–44]. Including more peripheral targets may be useful to make accurate inferences about response field properties beyond the examined range of reaching movements.

### Spatial response fields

We found strong evidence for a conjoint encoding of reach direction and amplitude; neurons in both areas exhibited significant modulation of movement planning activity by reach amplitude in some but not all reach directions. In particular, a dense sampling of reach targets revealed that neurons in both areas exhibit reliable spatial response patterns that are best predicted by a position-encoding model rather than a number of alternative direction and amplitude encoding models. Interestingly, this internal representation of the instructed reach target is highly consistent with previously observed sensory representations in the posterior parietal cortex [45–47], as well as corresponding response fields for saccadic movement plans.

The differences in model consistencies were not simply due to the complexity of models (i.e. the number of parameters), as models were evaluated in a cross-validation scheme. Importantly, the relatively high consistency of a Gaussian position-encoding model is not at odds with the relatively small proportions of neurons with significant modulation by amplitude, as compared to reach direction (~one third the population size). Indeed, for a large subset of neurons, both direction- and position-encoding models equally captured the neuronal response patterns (points along the unity line in Fig 7B and 7D), suggesting that this simple Gaussian model may concisely and accurately capture the response patterns of all (both amplitude-modulated and not) task-related neurons. Alternatively, it is possible that there exist two distinct populations of neurons that are best captured by two different models; however, our current data do not strongly suggest such heterogeneity.

## Conclusion

In this work, we characterized the responses of MIP neurons as monkeys planned and executed center-out reaches to targets varying in both direction and amplitude in the fronto-paralel plane. We found that a significant proportion of neurons had firing rate responses modulated by reach amplitude, but this population was relatively small (approximately one third in size) compared to reach direction. Moreover, these neurons were conjointly modulated by both reach direction and amplitude, in a manner consistent with an encoding of reach goals as a position in visual space. Indeed, the spatial response patterns of MIP neurons were best predicted by 2D Gaussian position encoding model, in contrast to a number of alternative direction and amplitude tuning models. Taken together, these results suggest that amplitude and direction jointly modulate activity in MIP to form representations of intended reach position.

## References

[1] Heath M, Neely K, Krigolson O, Binsted G, Elliott D. Memory-guided reaching: what the visuomotor system knows and how long it knows it. Vision and goal-directed movement: neurobahevioral perspectives Human Kinetics, Champaign. 2010:79–96.

[2] Georgopoulos AP, Kalaska JF, Caminiti R, Massey JT. On the relations between the direction of two-dimensional arm movements and cell discharge in primate motor cortex. The Journal of Neuroscience. 1982;2(11):1527–37. 7143039 10.1523/JNEUROSCI.02-11-01527.1982 PMC6564361

[3] Kalaska J, Caminiti R, Georgopoulos AP. Cortical mechanisms related to the direction of two-dimensional arm movements: relations in parietal area 5 and comparison with motor cortex. Experimental Brain Research. 1983;51(2):247–60. 6617794 10.1007/BF00237200

[4] Schwartz AB, Kettner RE, Georgopoulos AP. Primate motor cortex and free arm movements to visual targets in three-dimensional space. I. Relations between single cell discharge and direction of movement. The Journal of Neuroscience. 1988;8(8):2913–27. 3411361 10.1523/JNEUROSCI.08-08-02913.1988 PMC6569414

[5] Caminiti R, Johnson PB, Galli C, Ferraina S, Burnod Y, Urbano A. Making arm movements within different parts of space: the premotor and motor cortical representation of a coordinate system for reaching to visual targets. J Neurosci. 1991;11(5):1182–97. 2027042 10.1523/JNEUROSCI.11-05-01182.1991 PMC6575326

[6] Kakei S, Hoffman DS, Strick PL. Direction of action is represented in the ventral premotor cortex. Nature neuroscience. 2001;4(10):1020–5. doi: 10.1038/nn726 11547338 10.1038/nn726

[7] Heider B, Karnik A, Ramalingam N, Siegel RM. Neural representation during visually guided reaching in macaque posterior parietal cortex. Journal of neurophysiology. 2010;104(6):3494–509. doi: 10.1152/jn.01050.2009 20844104 10.1152/jn.01050.2009 PMC3007664

[8] Bremner LR, Andersen RA. Coding of the reach vector in parietal area 5d. Neuron. 2012;75(2):342–51. doi: 10.1016/j.neuron.2012.03.041 22841318 10.1016/j.neuron.2012.03.041 PMC3408621

[9] Cisek P, Kalaska JF. Simultaneous encoding of multiple potential reach directions in dorsal premotor cortex. Journal of Neurophysiology. 2002;87(2):1149–54. 11826082 10.1152/jn.00443.2001

[10] Fattori P, Kutz DF, Breveglieri R, Marzocchi N, Galletti C. Spatial tuning of reaching activity in the medial parieto-occipital cortex (area V6A) of macaque monkey. European Journal of Neuroscience. 2005;22(4):956–72. doi: 10.1111/j.1460-9568.2005.04288.x 16115219 10.1111/j.1460-9568.2005.04288.x

[11] Bhattacharyya R, Musallam S, Andersen RA. Parietal reach region encodes reach depth using retinal disparity and vergence angle signals. Journal of neurophysiology. 2009;102(2):805–16. doi: 10.1152/jn.90359.2008 19439678 10.1152/jn.90359.2008 PMC2724352

[12] Ferraina S, Brunamonti E, Giusti MA, Costa S, Genovesio A, Caminiti R. Reaching in depth: hand position dominates over binocular eye position in the rostral superior parietal lobule. The Journal of Neuroscience. 2009;29(37):11461–70. doi: 10.1523/JNEUROSCI.1305-09.2009 19759295 10.1523/JNEUROSCI.1305-09.2009 PMC6665750

[13] Hadjidimitrakis K, Bertozzi F, Breveglieri R, Bosco A, Galletti C, Fattori P. Common neural substrate for processing depth and direction signals for reaching in the monkey medial posterior parietal cortex. Cerebral Cortex. 2014;24(6):1645–57. doi: 10.1093/cercor/bht021 23382514 10.1093/cercor/bht021

[14] Hadjidimitrakis K, Dal Bo G, Breveglieri R, Galletti C, Fattori P. Overlapping representations for reach depth and direction in caudal superior parietal lobule of macaques. Journal of neurophysiology. 2015;114(4):2340–52. doi: 10.1152/jn.00486.2015 26269557 10.1152/jn.00486.2015 PMC4609758

[15] Piserchia V, Breveglieri R, Hadjidimitrakis K, Bertozzi F, Galletti C, Fattori P. Mixed body/hand reference frame for reaching in 3D space in macaque parietal area PEc. Cerebral Cortex. 2016:bhw039. 10.1093/cercor/bhw039 26941385

[16] Georgopoulos AP, Kettner RE, Schwartz AB. Primate motor cortex and free arm movements to visual targets in three-dimensional space. II. Coding of the direction of movement by a neuronal population. The Journal of Neuroscience. 1988;8(8):2928–37. 3411362 10.1523/JNEUROSCI.08-08-02928.1988 PMC6569382

[17] Riehle A, Requin J. Monkey primary motor and premotor cortex: single-cell activity related to prior information about direction and extent of an intended movement. Journal of Neurophysiology. 1989;61(3):534–49. 2709098 10.1152/jn.1989.61.3.534

[18] Fu Q, Suarez J, Ebner T. Neuronal specification of direction and distance during reaching movements in the superior precentral premotor area and primary motor cortex of monkeys. Journal of Neurophysiology. 1993;70(5):2097–116. 8294972 10.1152/jn.1993.70.5.2097

[19] Fu Q, Flament D, Coltz J, Ebner T. Temporal encoding of movement kinematics in the discharge of primate primary motor and premotor neurons. Journal of Neurophysiology. 1995;73(2):836–54. 7760138 10.1152/jn.1995.73.2.836

[20] Messier J, Kalaska JF. Covariation of primate dorsal premotor cell activity with direction and amplitude during a memorized-delay reaching task. Journal of Neurophysiology. 2000;84(1):152–65. 10899193 10.1152/jn.2000.84.1.152

[21] Kurata K. Premotor cortex of monkeys: set-and movement-related activity reflecting amplitude and direction of wrist movements. Journal of Neurophysiology. 1993;69(1):187–200. 8433130 10.1152/jn.1993.69.1.187

[22] Fabbri S, Caramazza A, Lingnau A. Distributed sensitivity for movement amplitude in directionally tuned neuronal populations. Journal of neurophysiology. 2012;107(7):1845–56. doi: 10.1152/jn.00435.2011 22205646 10.1152/jn.00435.2011

[23] Snyder L, Batista A, Andersen R. Coding of intention in the posterior parietal cortex. Nature. 1997;386(6621):167–70. doi: 10.1038/386167a0 9062187 10.1038/386167a0

[24] Seltzer B, Pandya D. Posterior parietal projections to the intraparietal sulcus of the rhesus monkey. Experimental Brain Research. 1986;62(3):459–69. 3720878 10.1007/BF00236024

[25] Shipp S, Blanton M, Zeki S. A visuo-somatomotor pathway through superior parietal cortex in the macaque monkey: cortical connections of areas V6 and V6A. European Journal of Neuroscience. 1998;10(10):3171–93. 9786211 10.1046/j.1460-9568.1998.00327.x

[26] Caminiti R, Genovesio A, Marconi B, Mayer AB, Onorati P, Ferraina S, et al Early coding of reaching: frontal and parietal association connections of parieto-occipital cortex. European Journal of Neuroscience. 1999;11(9):3339–45. 10510199 10.1046/j.1460-9568.1999.00801.x

[27] Lewis JW, Van Essen DC. Corticocortical connections of visual, sensorimotor, and multimodal processing areas in the parietal lobe of the macaque monkey. Journal of Comparative Neurology. 2000;428(1):112–37. 11058227 10.1002/1096-9861(20001204)428:1<112::aid-cne8>3.0.co;2-9

[28] Christopoulos VN, Bonaiuto J, Kagan I, Andersen RA. Inactivation of Parietal Reach Region Affects Reaching But Not Saccade Choices in Internally Guided Decisions. The Journal of Neuroscience. 2015;35(33):11719–28. doi: 10.1523/JNEUROSCI.1068-15.2015 26290248 10.1523/JNEUROSCI.1068-15.2015 PMC4540805

[29] Rajalingham R, Stacey RG, Tsoulfas G, Musallam S. Modulation of neural activity by reward in medial intraparietal cortex is sensitive to temporal sequence of reward. Journal of neurophysiology. 2014;112(7):1775–89. doi: 10.1152/jn.00533.2012 25008408 10.1152/jn.00533.2012 PMC4157169

[30] Friedman J, Hastie T, Tibshirani R. The elements of statistical learning: Springer series in statistics Springer, Berlin; 2001.

[31] Breveglieri R, Hadjidimitrakis K, Bosco A, Sabatini SP, Galletti C, Fattori P. Eye position encoding in three-dimensional space: Integration of version and vergence signals in the medial posterior parietal cortex. The Journal of Neuroscience. 2012;32(1):159–69. doi: 10.1523/JNEUROSCI.4028-11.2012 22219279 10.1523/JNEUROSCI.4028-11.2012 PMC6621321

[32] Messier J, Kalaska J. Differential effect of task conditions on errors of direction and extent of reaching movements. Experimental Brain Research. 1997;115(3):469–78. 9262201 10.1007/pl00005716

[33] Messier J, Kalaska JF. Comparison of variability of initial kinematics and endpoints of reaching movements. Experimental Brain Research. 1999;125(2):139–52. 10204767 10.1007/s002210050669

[34] Shen L, Alexander GE. Preferential representation of instructed target location versus limb trajectory in dorsal premotor area. Journal of Neurophysiology. 1997;77(3):1195–212. 9084590 10.1152/jn.1997.77.3.1195

[35] Seelke AM, Padberg JJ, Disbrow E, Purnell SM, Recanzone G, Krubitzer L. Topographic maps within Brodmann's area 5 of macaque monkeys. Cerebral Cortex. 2011:bhr257. 10.1093/cercor/bhr257 PMC3388892 21955920

[36] Lewis JW, Van Essen DC. Mapping of architectonic subdivisions in the macaque monkey, with emphasis on parieto-occipital cortex. Journal of Comparative Neurology. 2000;428(1):79–111. 11058226 10.1002/1096-9861(20001204)428:1<79::aid-cne7>3.0.co;2-q

[37] Krigolson O, Heath M. Background visual cues and memory-guided reaching. Human movement science. 2004;23(6):861–77. doi: 10.1016/j.humov.2004.10.011 15664677 10.1016/j.humov.2004.10.011

[38] Westwood DA, Heath M, Roy EA. No evidence for accurate visuomotor memory: Systematic and variable error in memory-guided reaching. Journal of motor behavior. 2003;35(2):127–33. doi: 10.1080/00222890309602128 12711584 10.1080/00222890309602128

[39] Sarlegna FR, Sainburg RL. The effect of target modality on visual and proprioceptive contributions to the control of movement distance. Experimental Brain Research. 2007;176(2):267–80. doi: 10.1007/s00221-006-0613-5 16896981 10.1007/s00221-006-0613-5 PMC10715720

[40] Brouwer A-M, Knill DC. The role of memory in visually guided reaching. Journal of Vision. 2007;7(5):6 doi: 10.1167/7.5.6 18217846 10.1167/7.5.6

[41] Batista AP, Buneo CA, Snyder LH, Andersen RA. Reach plans in eye-centered coordinates. Science. 1999;285(5425):257–60. 10398603 10.1126/science.285.5425.257

[42] Buneo CA, Jarvis MR, Batista AP, Andersen RA. Direct visuomotor transformations for reaching. Nature. 2002;416(6881):632–6. doi: 10.1038/416632a 11948351 10.1038/416632a

[43] Cui H, Andersen RA. Posterior parietal cortex encodes autonomously selected motor plans. Neuron. 2007;56(3):552–9. doi: 10.1016/j.neuron.2007.09.031 17988637 10.1016/j.neuron.2007.09.031 PMC2651089

[44] Gail A, Andersen RA. Neural dynamics in monkey parietal reach region reflect context-specific sensorimotor transformations. The Journal of neuroscience. 2006;26(37):9376–84. doi: 10.1523/JNEUROSCI.1570-06.2006 16971521 10.1523/JNEUROSCI.1570-06.2006 PMC6674591

[45] Andersen RA, Essick GK, Siegel RM. Encoding of spatial location by posterior parietal neurons. Science. 1985;230(4724):456–8. 4048942 10.1126/science.4048942

[46] Blatt GJ, Andersen RA, Stoner GR. Visual receptive field organization and cortico-cortical connections of the lateral intraparietal area (area LIP) in the macaque. Journal of Comparative Neurology. 1990;299(4):421–45. doi: 10.1002/cne.902990404 2243159 10.1002/cne.902990404

[47] Hamed SB, Duhamel J-R, Bremmer F, Graf W. Representation of the visual field in the lateral intraparietal area of macaque monkeys: a quantitative receptive field analysis. Experimental Brain Research. 2001;140(2):127–44. doi: 10.1007/s002210100785 11521146 10.1007/s002210100785

